# Cardiometabolic Risk Factors among Women with Eating Disorders in Saudi Arabia

**DOI:** 10.1155/2024/5953893

**Published:** 2024-06-05

**Authors:** Walaa Mohammedsaeed, Ahlam B. El Shikieri

**Affiliations:** ^1^Department of Clinical Laboratory Sciences, Faculty of Applied Medical Science at Taibah University, Madinah, Saudi Arabia; ^2^Department of Clinical Nutrition, College of Applied Medical Sciences, Taibah University, Medina, Saudi Arabia

## Abstract

**Objective:**

To assess selected cardiometabolic risk factors among Saudi women with eating disorders.

**Methods:**

An epidemiological, cross-sectional study included women aged between 18 and 50 years with eating disorders (EDs). Women with chronic diseases, pregnant, or lactating were excluded. The weight and height were measured for the calculation of the body mass index (BMI). Fasting blood samples were drawn for the analysis of blood sugar, glycated haemoglobin, lipid profile, albumin, haemoglobin, and C-reactive levels. The atherogenic dyslipidemia index (AIP) was also calculated.

**Results:**

Patients (*n* = 100) were enrolled. Fasting blood glucose levels were critically low among women with anorexia nervosa (AN) and bulimia nervosa (BN) but normal among those with binge eating disorders (BEDs). All women with ED suffered from anaemia based on their haemoglobin levels as well as dyslipidemia, hypoalbuminaemia, and high C-reactive protein levels. Women with AN had low cardiovascular (CV) risks based on their normal AIP values. However, women with BN and BED had intermediate CV risks. On average, women with AN suffered from severe thinness and those with BN had normal BMIs, whereas those with BED were overweight. Women (90%) with BN and BED were overweight and/or obese.

**Conclusion:**

Women with ED had a high risk of cardiovascular diseases defined by their hypoalbuminaemia, dyslipidemia, anaemia, and high AIP levels. Dietitians and psychiatrists are advised to collaborate in assessing the potential risk of having eating disorders to provide counselling sessions to women on healthy balanced diets and their effect on health.

## 1. Introduction

An eating disorder (ED) is a psychiatric illness described as a habitual disturbance of the attitude towards eating and weight control with diagnostic criteria based on psychological, behavioral, and physiological characteristics not related to medical or psychiatric factors [1]. The psychiatric conditions commonly coexisting with anorexia nervosa (AN), bulimia nervosa (BN), or binge eating disorders (BEDs) include major depressive disorder, bipolar disorder, panic, and anxiety disorders. ED is a serious, long-term health condition, which affects both men and women of all ages [2].

Studies have revealed that the prevalence of ED is increasing in low-middle-income countries such as non-Western countries, e.g., the Gulf countries and the Middle East region, as in high-income countries, and presents a serious health issue [2, 3]. Also, previously published work found that the risk of ED among women was found to be twice as prevalent as in men in countries such as Jordan, Libya, Palestine, and Syria [4, 5]. Women, in particular, are excessively concerned with their body image and weight and are very sensitive to their appearance, so they adopt dietary modifications that impose threats on their nutritional status and make them more vulnerable to ED.

Moreover, ED can have long-lasting effects on overall health, one of which is reproductive health, altering the menstrual function, delaying child-bearing or infertility, and leading to pregnancy complications [6–8]. Furthermore, a cross-sectional study conducted in Dammam, Kingdom of Saudi Arabia, found media and advertisement exposure, external pressure from peers, increased focus on having a thin body shape, and having low grades at university to be risk factors for ED among female students aged 18–23 years [9]. Also, cardiometabolic risk factors (CMRFs) and metabolic syndrome (MetS) are known to be directly associated with nutrition [5]. CMRFs show an accelerating trend in most developing countries and are becoming significant contributors to the burden of diseases, death, and disability [10, 11]. Evidence suggests that nutritional transition is associated with the increasing trend in CMRFs. Globally, limited studies addressed the risk of cardiovascular diseases (CVDs) among patients with eating-disordered behaviors. Therefore, the current study aimed to assess selected CMRFs among women with ED. It is hypothesized that Saudi women with ED are at increased risk of CVD.

## 2. Materials and Methods

An epidemiological, cross-sectional study, including students and employees, was conducted from January to March 2019 at Taibah University. Two thousand females (*n* = 2000) were recruited from various sectors at Taibah University, including both students (*n* = 1767) and employees (*n* = 233). Women aged 18–50 years were targeted. Pregnant and lactating women, those with upper and lower gastrointestinal tract disease, and sufferers of chronic diseases that might affect eating habits, such as diabetes, hypertension, or thyroid disorders, were excluded. In addition, women using hormonal contraception, such as oral contraceptive pills, were excluded.

A systematic random sampling technique was followed to recruit the study population. The sample size was determined based on the following equation:

(1) $$\begin{array}{c} n={\left(Z_{1-\sigma /2}\right)}^{2}\times P\left(1-P\right)÷d^{2},c\text{onsidering }a\text{ confidence level of }95\%,\;Z_{1-\sigma /2}=1.96,d=0.05. \end{array}$$

**Figure 1:**
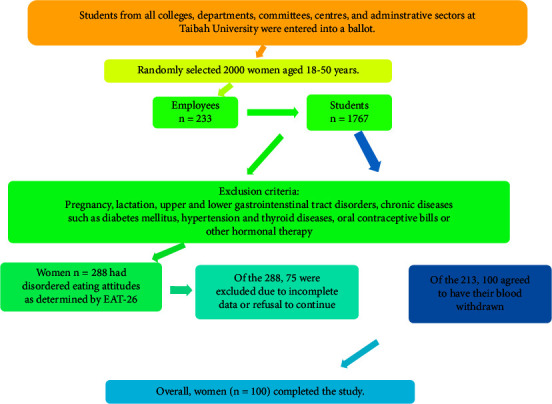
Flowchart for the selection of women.

First, a list of the different colleges, committees, centres, and administrative units were obtained from the official website of Taibah University (https://www.taibahu.edu.sa). They were then entered into a ballot to select the sites randomly. Finally, the required number of participants was selected proportionally according to the total number of students and employees, as shown in Figure 1.

### 2.1. Defining and Diagnosing ED

#### 2.1.1. The Eating Attitudes Test (EAT-26)

This test was used to determine women with a risk of disordered eating. It helps identifying ED tendencies among people in clinical and nonclinical settings. The tool has been previously validated in a study conducted in Saudi Arabia [12]. It has the following three sets of questions: In Part A, information about current and ideal body weight was obtained; Part B included three subscales: dieting, bulimia and food preoccupation, and oral control, were questioned with a total of 26 questions. Each item (except the 26th item) had six response options ranging from 0 to 3. Finally, in part C, four behavioral questions aimed to determine the extreme weight control and estimate their frequency, e.g., self-induced vomiting over the preceding six months. Overall, females who scored ≥20 on the EAT-26 were classified as “at risk” for ED [13, 14].

#### 2.1.2. Diagnostic Statistical Manual of Mental Disorders, Fifth Edition (DSM-5)

After the EAT-26, all women diagnosed with ED completed the DSM-5, which is a 23-component self-reported questionnaire that identifies ED, namely, AN, BN, BED, OSFED, and UFED. The DSM-5 criteria identify more individuals within specified diagnoses, reducing rates of other or unspecified diagnoses. It captures the range of ED psychopathology [15]. Anorexia nervosa, BN, and BED are the typical ED, whereas OSFED and UFED are known as atypical forms of ED [16]. The OSFED includes the atypical AN, low‐frequent BN, low‐frequent BED, night eating syndrome (NES), and purging disease (PD). The DSM-5 questionnaire starts with questions about physical appearance and how shape influenced judgment as a person. Scores ranged from zero to six, where zero means not at all (not suffering from this problem) and six means extremely (highly suffering from the problem). Then, questions about having episodes of eating with a loss of control and feelings through and after overeating, e.g., eating much and more rapidly, were asked. Moreover, DSM-5 included information about the highest weights at the current height. The current study only included women with ED. The latter were included in the study after the aims were clearly explained. Those who agreed to participate signed a consent form before the start of the study, which stated that they were free to withdraw from the study without prior notice and their privacy would be respected (Figure 1).

### 2.2. Specimen Collection and Laboratory Analysis

Fasting blood samples (3 ml) were drawn from women to analyze selected parameters, including lipid profile concentrations. The serum was separated from each sample by centrifuging at 3000 rpm for five minutes to estimate the biomarker profiles. Trained female nurses drew blood samples after women fasted for at least 8 hours overnight. Blood specimens were stored in a freezer at −70°C until testing. The CMRF blood markers were determined using immunoassay technology using the Cobas b 311 immunoassay analyzer according to the manufacturer's instructions (Roche Diagnostics, GmbH, Germany). Fasting blood sugar (FBS), glycated haemoglobin (HbA1c), total cholesterol (TC), high-density lipoprotein (HDL-C), low-density lipoprotein (LDL-C), and triglycerides (TG) were measured enzymatically.

In addition, plasma ferritin, albumin, and C-reactive protein (CRP) levels were measured using chemiluminescence. Haemoglobin (Hb) was immediately measured in the field with a drop of whole blood using a HemoCue® (Hemocue, Sweden). Serum insulin was used to determine the hormonal insulin level by the chemiluminescent enzyme-labeled immunometric assay using an IMMULITE 2000 Systems analyzer (Siemens, Gwynedd, UK) according to the manufacturer's directions. The serum insulin concentration was measured, and the homeostasis model assessment (HOMA) equation (fasting glycaemia × serum insulin)/22·5) was used as an index of insulin resistance; insulin resistance was defined as HOMA-IR ≥2.9. The cut-offs used for the various biomarkers are shown in Table 1. Furthermore, the atherogenic dyslipidemia index (AIP) was further calculated as log_10_ (TG/HDL-C) as a predictor for cardiovascular diseases. An AIP value of less than 0.11 depicts a low risk of CVD; values between 0.11 and 0.21 depict an intermediate risk, while values above 0.21 are associated with a high risk of CVD (Table 1).

#### 2.2.1. Cardiometabolic Risk Factors (CMRFs)

Conventional risk factors for cardiovascular disease (CVD) encompass age, BMI, and dyslipidemia (as per the National Cholesterol Education Program (NCEP) criteria: increased cholesterol, increased triglycerides, elevated LDL-C levels, and reduced HDL-C). An assessment was conducted to investigate if insulin resistance (IR), inflammation measured by high-sensitivity C-reactive protein (hs-CRP) levels, and AIP (log10TG/HDL-C) may serve as indicators of atherogenic dyslipidemia, a newer cardiovascular risk factor.

### 2.3. Anthropometric Measurements

After calibrating the scales, the weight and height were measured twice using an electronic scale (Beurer GmbH Type PS 07, China). Measurements were taken twice during data collection and not on different days. The body mass index (BMI) was calculated using standard formulas (weight in kilogram ÷ height in meters square). BMI classifications used were underweight <18.5, normal = 18.5–25, overweight = 25–29.9, and obese ≥30 [17] (STEPS Surveillance Manual. Geneva: World Health Organization; 2008).

### 2.4. Statistical Method

Statistical analysis was performed using GraphPad Prism 7 (GraphPad Software, CA, USA). Quantitative data were expressed as mean ± SD. Both mean and number of women with normal and elevated values were included. The mean was used to summarize and represent the dataset of the sample of women included. It was used as a measure for central tendency. The percentages were calculated to assess the levels of key variables in patients with ED. The correlation between the CMRFs was determined using Pearson's correlation. A two-way ANOVA with a post hoc Tukey test was utilized to compare the different ED groups. Multivariate analysis was used based on the multiple linear regression, and the unstandardized coefficients (B) and odds ratios (ORs) were calculated with 95% confidence intervals (CIs). We did the Chi-square test to determine differences between study groups based on the number of CMRFs as categorized by the types of ED. All differences were statistically significant at the level of *P* ≤ 0.05. Ethical considerations: ethical approval to conduct the study was obtained from the Ethical Committee at the College of Applied Medical Sciences, Taibah University, Al Madinah Al Munawarah (Ethical clearance number: CLN201813).

## 3. Results

### 3.1. Cardiometabolic Risk Factors among Women with ED as Categorized by Their ED Types

Out of the 213 women with ED, only 100 agreed to have their blood samples drawn to identify CMRFs. The remaining 113 women were referred to the Medical Centre at the University for further investigations since they refused to have their blood samples withdrawn.  Table 2 presents the distribution of women according to age, with a focus on two groups: those between 20 and 30 years old, who were predominantly students, and those beyond 30 years old, who were primarily employees. Of the 100 women included, 18% had AN, 32% had BN, and 50% had BED. The average FBG level was severely low among women with AN and BN, whereas it was normal among those with BED. In addition, many women with AN suffered from hypoglycemia compared to their counterparts (Table 2). Also, there were no significant differences among women regarding their HA1C, insulin, and insulin resistance levels, which were within desirable levels. Moreover, all women suffered from anaemia as defined by their low Hb levels. However, the mean serum ferritin was within normal levels for women with BN levels although more than a third suffered from low ferritin levels (Table 2).

Moreover, all women had dyslipidemia. On average, they had low fasting HDL-C, high LDL-C, TC, and TAG levels. Also, their TC:HDL-C ratio was high (Table 2). In addition, women suffered from hypoalbuminaemia; women with AN were the most affected. Besides, all women had higher CRP levels. The most affected women were those who had BED (Table 2).

Furthermore, the average AIP indicated that women with AN had low CVD risk, whereas those with BN and BED had intermediate risk. More than 90% of the women with BN and BED had intermediate to high CVD risks (Table 2). Also, on average, women with AN were classified as having severe thinness, whereas those with BN had normal BMI, and those who suffered from BED were overweight. Many women with BN and BED (90%) were overweight and/or obese (Table 2).

In addition, many women had 2–3 CMRFs. Women with various types of ED had a comparable number of CMRFs. For instance, women with BN had 2–3 risk factors, whereas women with BED had >4 CMRFs (Table 3).

### 3.2. Correlation among the CMRFs for Women with ED

Pearson correlation revealed that among women with AN, BN, and BED, there were significant associations among CMRFs (Table 4). Similar trends were seen among women with ED. For instance, TC correlated positively with TAG, CRP, AIP, and BMI. Also, TAG directly correlated with FBG, CRP, AIP, and BMI. Serum ferritin was positively associated with albumin among women with ED. The CRP tended to increase significantly with AIP (*P*=0.05) among women with BED. In addition, CRP was inversely correlated with FBG, irrespective of the type of ED (Table 4).

### 3.3. Multiple Linear Regression Analysis

The results revealed that the various types of ED (dependent variable) predicted CMRFs as determined by the multiple linear regression model. Women with AN had a higher risk of hypoglycemia, anaemia, hypoalbuminaemia, and thinness (Table 5). Moreover, women with BN and BED had an increased risk of hypoglycemia (BN only), anaemia, hypoalbuminaemia, dyslipidemia, elevated CRP, AIP, and higher body weights (Table 5).

## 4. Discussion

Cardiometabolic risk factors are accelerating in many countries and are becoming significant contributors to the burden of diseases, death, and disability [10, 11]. The larger the number of CVD risk factors, such as obesity, diabetes, hypertension, and hyperlipidemia, the greater the odds of CVD [18]. In addition, the CVD risk is associated with people's eating patterns, especially ED [19]. Our study aimed to assess selected CMRFs among women with ED.

Findings revealed that hypoglycaemia was common among women with AN and BN. Low fasting glucose levels are a common medical complication among AN patients, mainly due to dietary restriction and starvation [20]. Women with AN in our study had very low fasting glucose levels compared to their counterparts in Naples previously surveyed by Omodei and coworkers (1.4 versus 3.76 mmol/L, respectively) [21]. Lower glucose levels were also realized compared to other studies [22]. A meta-analysis revealed that hypoglycaemia is associated with blood coagulation, cell adhesion, and inflammatory markers [23]. These complications adversely affect the endothelial function and blood flow, increasing the risk of atherosclerosis. Our study further showed a negative correlation between FBG levels and CRP, a surrogate inflammatory marker [24, 25].

Furthermore, our study revealed that all women with various EDs suffered from anaemia. The prevalence of women with AN who had anaemia was nearly double their counterparts in Massachusetts previously researched by Miller and co-workers (72% versus 39%, respectively) [26]. Also, compared to the Australian women with AN and BN, patients in the current study had very low serum ferritin levels (AN = 68.2 versus 14; BN = 46 versus 21 *μ*g/L, respectively) [27].

Our study showed that women with BN had very low haemoglobin levels compared to their counterparts in previously published studies (8.7 versus 12.7 g/dl) [28]. Anaemia is one of the nontraditional risk factors for CVD [29]. Anaemia results in decreased blood viscosity, increased venous return, augmented preload, and activated sympathetic nervous systems, which ultimately cause an increased heart rate and stroke volume [30]. The latter raised the cardiac output, which could lead to left ventricular enlargement and eccentric left ventricular hypertrophy, which may aggravate ischemic heart disease [31].

Although our study revealed that women with AN were severely underweight, their BMI (17.8 kgm^−2^) was higher than their counterparts in the outpatient clinics in other countries, such as Massachusetts (16.9 kgm^−2^) [26] and Naples (15.95 kgm^−2^) [21]. Similar findings were shown among Australian patients. It is important to note that the women in our study were not previously diagnosed with ED and were not on active treatment for the condition. In the current study, women with AN had higher BMI (17.8 kgm^−2^ versus 16.5 kgm^−2^), whereas those with BN had similar BMIs (20.5 kgm^−2^) than their Australian counterparts [27]. Previous studies indicated that severely undernourished AN patients suffered from subclinical myocardial impairments, which could be fatal [32].

Contradictory to our study findings, women with BN had lower BMI than reported in other studies [28]. Moreover, the prevalence of overweight and obese women with BN was higher than in the World Health Organization World Mental Health (WHO WMH) Survey (overweight = 43.8 versus 32.4% and obesity = 46.8 versus 32.8%, respectively). On the other hand, the number of obese women with BED in our study (30%) was lower than their counterparts in the WHO WMH (36.2%) although overweight was more prevalent in the current study (60 versus 30.7%) [33, 34]. In our study, BMI correlated positively with TC and TAG among women with ED, and BMI was directly associated with fasting glucose levels and AIP among women with BED. Obesity is a traditional chronic disease, which is associated with CVD and dyslipidemia [35]. In the Framingham Heart Study, patients with subjective (0.9%) and objective (1.6%) BED had a higher 10-year risk of the CVD event, with most of the risk being mediated by the increased body weight [36]. In addition, stigmatization or discrimination against overweight individuals increases the risk of stress, low self-esteem, depression, loneliness, weight gain, and suicide attempts [37]. Also, weight stigma damages health, undermines human and social rights, and is unacceptable in modern societies [38]. Different studies use various BMI classifications, and thus, comparisons should be noted cautiously.

Our study showed that the prevalence of hypoalbuminaemia among women with AN was 4.6 times higher in our study (83%) than among American women (18%) [26]. In addition, Australian women with AN and BN had better albumin levels than our study groups (*N* = 46.3 versus 20 and BN = 45.5 versus 14.2 g/L, respectively) [27]. Similar findings were reported elsewhere [28]. Albumin decreases slowly in response to nutritional stress due to its large extravascular pool and long half-life among BN patients [28]. Traditionally, hypoalbuminaemia is linked to malnutrition and inflammation. However, recent studies revealed the association between the low serum albumin and CVD risk, such as heart failure [28–39]. This may be related to the role of albumin in the body, such as being an anti-inflammatory, the most important antioxidant, and anticoagulant [39].

Additionally, in the current study, dyslipidemia was found to be a common phenomenon among women with ED. Moreover, our study revealed that hypercholesterolemia was prevalent among women with BN and BED although all women with ED suffered from high serum cholesterol levels. In Naples, women with AN had lower mean TC (4.4 versus 6.1 mmol/L) and higher HDL-C (1.66 versus 0.99 mmol/L) [21] than women in our study. In addition, women with AN in our study had lower TC (6.1 versus 10.6 mmol/L), TAG (3.1 versus 5.52 mmol/L), and HDL-C (0.99 versus 3.98 mmol/L) than their Spanish counterparts [22]. In our study, hypercholesterolemia was prevalent among 62.5% of women with BN, which is far higher than what was previously published. Our study had higher mean serum cholesterol levels than the Australian women with AN and BN (AN = 4.72 versus 6.1 and BN = 4.59 versus 7.8 mmol/L, respectively) [27]. Similar findings were also published elsewhere [40]. In our study, TC increased significantly with TAG, CRP, and AIP, all independent CVD markers.

Moreover, BN is linked to metabolic alterations that might elevate the likelihood of chronic nonreversible cardiovascular diseases (CVDs). One of these alterations is the lipid profile [41]. Previously published studies revealed that the proportion of women diagnosed with BN who have hypercholesterolemia ranges from 19% to 48% [42]. Bulimia was associated with 21.93 (95% CI, 9.29–51.74) times the risk of myocardial infarction at two years and 14.13 (95% CI, 6.02–33.18) times the risk at five years [43]. Dyslipidemia is a traditional risk factor for CVD [44], which is prevalent among women in the current study.

CRP is an acute-phase protein that is recognized as a predictor, pathogenic, and new marker of cardiovascular disease (CVD) [45]. It also plays a vital role in atherogenesis [46]. Our study revealed that all the women with ED had higher-than-normal CRP values, with those suffering from BED being the most affected. As previously reported, our study showed that CRP correlated positively with TC and TAG [46]. Unlike other studies, CRP correlated negatively with FBG [46]. In our study, women with AN had higher CRP levels than their counterparts in other studies (21 versus 0.54 mg/L) [47].

In addition, our investigation showed that all women with erectile dysfunction exhibited heightened levels of AIP. Many women with BN and BED suffered from intermediate and high CVD risk, as determined by their high AIP. The AIP correlated positively with TC and TAG among all patients. The atherogenic index of plasma is a critical index, which could be used as a stand-alone index for cardiac risk estimation [48]. The index predicts atherosclerosis and CVD more strongly than the traditional atherogenic lipid profile [49].

Although EDs are associated with several CVD complications, which could be fatal, we did not find any published studies in Saudi Arabia that determined the potential risk of CMRF among these vulnerable patients. Our study was considered the first of its kind, which assessed the cardiometabolic risk among women with eating-disordered behaviors. It could act as a basis for future studies. The study also screened a significant number of women (*n* = 1700), which is considered a large sample compared to published studies that determined the prevalence of ED. Also, the study did not focus only on students as in other studies but included employees who are usually not studied explicitly. However, some of the drawbacks of our current study are the study design, sampling method, and the inclusion of one sex only, i.e., women. For instance, the cross-sectional study design limits causal inferences. It would be ideal to follow women with CMRFs to determine CVD events. In addition, experimental studies that evaluate the influence of dysfunctional eating patterns on the development of metabolic risk factors for CVD are other ways to determine the causality between these dysfunctional eating patterns and the metabolic risk and to uncover potential mechanisms that may explain these associations. Moreover, interventions are needed to reduce ED in the context of CVD risk factor reduction.

In addition, men were not included in the study. Thus, the findings need to be interpreted with caution. Also, blood samples were drawn only once. Therefore, the bias added due to the effect of the day-to-day variation should not be neglected. However, it was impossible to draw second blood samples since women with ED had a phobia of drawing blood, which was noticed by the small number of women who agreed to participate in the study.

In conclusion, our findings suggest that Saudi women with AN, BN, and BED suffer from multiple CMRFs, both traditional and nontraditional. Therefore, the Medical Unit Authority at Taibah University should launch routine health intervention programs to increase awareness about the importance of healthy dietary patterns, signs and symptoms of ED, and the risks associated with developing ED. Annual screening for ED among students and employees is highly recommended. In addition, these intervention programs should be composed of a multidisciplinary team, including physicians, nurses, dieticians, and social workers. Patients with eating disorders should be contacted, and their CMRFs should be determined, treated, and followed up.

## Figures and Tables

**Table 1:** The normal reference range for CMRFs among women.

Parameters	Normal reference range for women
HbA1c (%)	4.3–6.0
FBG (mmol/L)	3.89–5.50
Insulin (pmol/L)	<174
Insulin resistance (IR)	<2.9
Serum ferritin (ng/mL)	18–160
Haemoglobin (g/dl)	12–16
Serum albumin (g/L)	30–50
HDL-C (mmol/L)	1.04–1.55
LDL-C (mmol/L)	2.59–4.11
TC (mmol/L)	5.2–6.2
TAG (mmol/L)	1.7–2.2
TC: HDL-C	<5
CRP (mg/l)	<10
AIP	<0.11
BMI (kg/m^2^)	18.5–24.9

Values are obtained from references (references range acquired from Madinah Hospital labs in Madinah region, Saudi Arabia).

**Table 2:** Biomarker levels, AIP, and BMI of women with ED. Results were categorized based on the types of ED.

Risk factors	Total women (*n*=100)	AN (*N*=18)	BN (*N*=32)	BED (*N*=50)	*P* value
*Age*					
20–30 years	25 ± 9^a^	20 ± 10^a^	22 ± 8^a^	23 ± 11^a^	**>0.05**
	55 (55)^b^	10 (55.5)^b^	17 (53.2)^b^	28 (56)^b^	
31–50 years	35 ± 10^a^	35 ± 10^a^	34 ± 12^a^	40 ± 9^a^	
	45 (45)^b^	8 (44.5)^b^	15 (46.8)^b^	22 (44)^b^	

*Fasting glucose levels*					
Mean ± SD	2.8 ± 0.92^a^	1.4 ± 0.34	2.1 ± 0.43	5.4 ± 0.34	**0.02** ^*∗*^
Low: <3.9	25 (25)^b^	10 (55.5)	5 (15.6)	10 (20)	
Normal: 3.9–5.5	75 (75)	8 (44.4)	27 (84.4)	40 (80)	

*HA1C%*					
Mean ± SD	5.4 ± 0.3^a^	4.9 ± 0.24	5.1 ± 0.21	5.3 ± 0.4	>0.05
Low: <4.3	7 (7)^b^	0	2 (6.3)	5 (10)	
Normal: 4.3–6.0	92 (92)	18 (100)	30 (93.8)	44 (88)	
High: >6	1 (1)	0	0	1 (2)	

*Insulin level*					
Mean ± SD	180.0 ± 20.0^a^	170 ± 22.0	172.6 ± 21.2	172.8 ± 23.4	>0.05
Low: <111	7 (7)^b^	0	2 (6.3)	5 (10)	
Normal: <174	93 (93)	18 (100)	30 (93.8)	44 (88)	
High: >1917	1 (1)	0	0	1 (2)	

*Insulin resistance*					
Mean ± SD	0.99 ± 0.6^a^	0.98 ± 0.8	1.1 ± 0.9	1.3 ± 0.7	>0.05
Normal: <2.9	93 (93)^b^	18 (100)	30 (93.8)	44 (88)	
High: >2.9	1 (1)	0	0	1 (2)	

*Serum ferritin*					
Mean ± SD	16.1 ± 10.73^a^	14 ± 9.45	21 ± 13.36	16 ± 8.17	**0.02** ^*∗*^
Low: <18	60 (60)^b^	11 (61.1)	12 (37.5)	35 (70)	
Normal = 18–160	40 (40)	7 (38.9)	20 (62.5)	15 (30)	

*HB*					
Mean ± SD	5.4 ± 0.33^a^	7.6 ± 5.83	8.7 ± 6.92	7.8 ± 6.66	>0.05
Low: <12	38 (38)^b^	13 (72.2)	10 (31.3)	15 (30)	
Normal: 12–16	62 (62)	5 (27.8)	22 (68.8)	35 (70)	

*HDL-C*					
Mean ± SD	0.89 ± 0.59^a^	0.99 ± 0.15	0.95 ± 0.21	0.87 ± 0.12	**0.05** ^*∗*^
Low: <1.03	63 (63)^b^	7 (38.9)	23 (71.9)	33 (66)	**0.04** ^*∗*^
Normal: >1.55	37 (37)	11 (61.1)	9 (28.1)	17 (34)	

*LDL-C*					
Mean ± SD	4.79 ± 0.75^a^	4.2 ± 7.3	4.8 ± 6.4	6.3 ± 2.5	**0.001** ^*∗∗*^
High: >4.11	65 (65)^b^	5 (27.8)	25 (78.1)	35 (70)	
Normal: <3.36	35 (35)	13 (72.2)	7 (21.9)	15 (30)	

*Total cholesterol*					
Mean ± SD	7.1 ± 2.23^a^	6.1 ± 2.15	7.8 ± 2.25	9.99 ± 3.13	**0.04** ^*∗*^
High: >6.2	61 (61)^b^	6 (33.3)	20 (62.5)	35 (70)	**0.03** ^*∗*^
Normal: <5.2	39 (39)	12 (66.7)	12 (37.5)	15 (30)	

*TAG*					
Mean ± SD	3.11 ± 1.59^a^	3.1 ± 1.23	4.2 ± 3.24	5.5 ± 1.35	**0.04** ^*∗*^
High: >2.25	68 (68)^b^	8 (44.4)	23 (71.9)	37 (74)	**0.02** ^*∗*^
Normal: <1.69	32 (32)	10 (55.6)	9 (28.1)	13 (26)	

*TC: HDL-C*					
Mean ± SD	6.9 ± 1.75^a^	6.03 ± 0.99	6.3 ± 0.95	6.8 ± 1.59	**0.01** ^*∗*^
Normal: <5	5 (5)^b^	4 (22.2)	1 (3.1)	—	
High: >5	95 (95)	14 (77.8)	31 (96.9)	50 (100)	

*Albumin*					
Mean ± SD	23.7 ± 11.8^a^	20.1 ± 10.23	14.2 ± 8.24	13.5 ± 10.35	**0.03** ^*∗*^
Low: <30	39 (39)^b^	15 (83.3)	11 (34.4)	13 (26)	**0.02** ^*∗*^
Normal: 30–50	61 (61)	3 (16.7)	21 (65.6)	37 (74)	

*CRP*					
Mean ± SD	20 ± 10.3^a^	21 ± 10.2	22 ± 15.3	29 ± 12.5	**0.03** ^*∗*^
Normal: <10	46 (46)^b^	10 (55.5)	17 (53.1)	19 (38)	
High: >10	54 (54)	8 (44.5)	15 (46.9)	31 (62)	

*AIP*					
Mean ± SD	0.21 ± 0.1	0.11 ± 0.11	0.20 ± 0.12	0.23 ± 0.20	**0.02** ^*∗*^

*AIP classifications*					
Low risk: <0.11	12 (12)	9 (50)	2 (6.3)	1 (2)	**0.01** ^*∗*^
Intermediate risk = 0.11–0.21	54 (54)	9 (50)	16 (50)	29 (58)	
High risk: >0.21	34 (34)	—	14 (43.7)	20 (40)	

*BMI*					
Mean ± SD	17 ± 2.21	17.8 ± 4.7	20.7 ± 3.4	25.9 ± 4.8	**0.02** ^*∗*^

*BMI classifications*					
Underweight <18.5	16 (16)	16 (88.9)	—	—	**0.001** ^*∗∗*^
Normal = 18.5–24.9	10 (10)	2 (11.1)	3 (9.4)	5 (10)	
Overweight = 25–29.9	44 (44)	—	14 (43.8)	30 (60)	
Obesity ≥30	30 (30)	—	15 (46.8)	15 (30)	

^a^Values are mean ± standard errors; ^b^values are number and percentages; AN = anorexia nervosa; BN = bulimia nervosa; BED = binge eating disorder; *P* values were obtained from two-way ANOVA test with post hoc; ^*∗*^statistical differences between AN and BN; ^*∗∗*^statistical differences between AN and BED. Bold values indicates the significance values.

**Table 3:** Number of CMRFs as categorized by the types of ED.

Number of CMRFs	Total women *n* (%)	AN *n* (%)	BN *n* (%)	BED *n* (%)	*P* value
0-1	10 (10)	1 (5.5)	5 (15.6)	4 (8)	**0.04** ^*∗*^
2-3	55 (55)	10 (55.6)	20 (62.5)	25 (50)	
>4	35 (35)	7 (38.9)	7 (21.8)	21 (42)	

*P* value from Chi-square; values are numbers and percentages. P: probability, comparing the difference in the number of CMRFs in different types of ED, ^*∗*^: significant. *P* ≤ 0.05^*∗*^, ≤0.001^*∗∗*^. Bold values indicates the significance values.

**Table 4:** The correlations between CMRFs among women with ED.

CMRFs	FBG		TC		TAG		CRP		BMI		Ferritin		AIP		Albumin	
	*r*	*P*	*r*	*P*	*r*	*P*	*r*	*P*	*r*	*P*	*r*	*P*	*r*	*P*	*r*	*P*
*AN patients*																
FBG	1	—	0.21	0.07	0.47	**0.03** ^*∗*^	−0.39	**0.04** ^*∗*^	0.35	0.07	0.30	>0.05	0.31	>0.05	0.40	>0.05
TC	0.21	0.07	1	—	0.49	**0.02** ^*∗*^	0.58	**0.01** ^*∗*^	0.97	**0.01** ^*∗*^	0.22	0.07	0.53	**0.04** ^*∗*^	0.55	**0.05** ^*∗*^
TAG	0.47	**0.03** ^*∗*^	0.49	**0.02** ^*∗*^	1	—	0.46	**0.05** ^*∗*^	0.73	**0.04** ^*∗*^	0.42	>0.05	0.74	**0.02** ^*∗*^	0.33	0.06
CRP	−0.39	**0.04** ^*∗*^	0.58	**0.01** ^*∗*^	0.46	**0.05** ^*∗*^	1	—	0.22	0.08	0.58	**0.04** ^*∗*^	0.36	0.08	0.84	**0.03** ^*∗*^
BMI	0.35	0.07	0.97	**0.01** ^*∗*^	0.73	**0.04** ^*∗*^	0.22	0.08	1	—	0.11	0.09	0.31	>0.05	0.10	>0.05
Ferritin	0.30	>0.05	0.22	0.07	0.42	>0.05	0.58	**0.04** ^*∗*^	0.11	0.09	1	—	0.25	0.06	0.52	**0.04** ^*∗*^
AIP	0.31	>0.05	0.53	**0.04** ^*∗*^	0.74	**0.02** ^*∗*^	0.36	0.08	0.31	>0.05	0.25	0.06	1	—	0.12	0.07
Albumin	0.40	>0.05	0.55	**0.05** ^*∗*^	0.33	0.06	0.84	**0.03** ^*∗*^	0.10	>0.05	0.52	**0.04** ^*∗*^	0.12	0.02	1	—

*BN patients*																
FBG	1	—	0.21	0.06	0.59	**0.03** ^*∗*^	−0.56	**0.02** ^*∗*^	0.54	**0.04** ^*∗*^	0.34	>0.05	0.33	>0.05	0.42	>0.05
TC	0.21	0.06	1	—	0.53	**0.02** ^*∗*^	0.59	**0.01** ^*∗*^	0.88	**0.01** ^*∗*^	0.25	0.07	0.55	**0.04** ^*∗*^	0.59	**0.05** ^*∗*^
TAG	0.59	**0.03** ^*∗*^	0.53	**0.02** ^*∗*^	1	—	0.49	**0.05** ^*∗*^	0.74	**0.04** ^*∗*^	0.44	>0.05	0.79	**0.02** ^*∗*^	0.33	0.06
CRP	−0.56	**0.02** ^*∗*^	0.59	**0.01** ^*∗*^	0.49	**0.05** ^*∗*^	1	—	0.30	0.08	0.59	**0.04** ^*∗*^	0.37	0.08	0.84	**0.03** ^*∗*^
BMI	0.54	0.04^*∗*^	0.88	**0.01** ^*∗*^	0.74	**0.04** ^*∗*^	0.30	0.08	1	—	0.23	0.09	0.38	>0.05	0.14	>0.05
Ferritin	0.34	>0.05	0.25	0.07	0.43	>0.05	0.59	**0.04** ^*∗*^	0.23	0.09	1	—	0.23	0.06	0.54	**0.04** ^*∗*^
AIP	0.33	>0.05	0.55	**0.04** ^*∗*^	0.79	**0.02** ^*∗*^	0.37	0.08	0.38	>0.05	0.23	0.06	1	—	0.32	0.07
Albumin	0.42	>0.05	0.59	**0.05** ^*∗*^	0.33	0.06	0.84	**0.03** ^*∗*^	0.14	>0.05	0.54	**0.04** ^*∗*^	0.32	0.07	1	—

*BED patients*																
FBG	1	—	0.20	0.06	0.54	**0.03** ^*∗*^	−0.70	**0.02** ^*∗*^	0.66	**0.03** ^*∗*^	0.32	>0.05	0.30	>0.05	0.42	>0.05
TC	0.20	0.06	1	—	0.57	**0.02** ^*∗*^	0.68	**0.01** ^*∗*^	0.89	**0.01** ^*∗*^	0.11	0.07	0.57	**0.04** ^*∗*^	0.59	**0.05** ^*∗*^
TAG	0.54	**0.03** ^*∗*^	0.57	**0.02** ^*∗*^	1	—	0.59	**0.05** ^*∗*^	0.69	**0.04** ^*∗*^	0.41	>0.05	0.77	**0.02** ^*∗*^	0.44	0.06
CRP	−0.70	**0.02** ^*∗*^	0.68	**0.01** ^*∗*^	0.59	**0.05** ^*∗*^	1	—	0.26	0.08	0.65	**0.04** ^*∗*^	0.45	**0.05** ^*∗*^	0.89	**0.03** ^*∗*^
BMI	0.66	**0.03** ^*∗*^	0.89	**0.01** ^*∗*^	0.69	**0.04** ^*∗*^	0.26	0.08	1	—	0.20	0.09	0.54	**0.05** ^*∗*^	0.21	>0.05
Ferritin	0.32	>0.05	0.11	0.07	0.41	>0.05	0.65	**0.04** ^*∗*^	0.20	0.09	1	—	0.28	0.06	0.59	**0.04** ^*∗*^
AIP	0.30	>0.05	0.57	**0.04** ^*∗*^	0.77	**0.02** ^*∗*^	0.45	**0.05** ^*∗*^	0.54	**0.05** ^*∗*^	0.28	0.06	1	—	0.12	0.07
Albumin	0.42	>0.05	0.58	**0.05** ^*∗*^	0.44	0.06	0.89	**0.03** ^*∗*^	0.21	>0.05	0.59	**0.04** ^*∗*^	0.12	0.07	1	—

*P* values were obtained from Pearson's correlation; starred values indicate significant levels. *P* ≤ 0.05^*∗*^, ≤0.001^*∗∗*^. Bold values indicates the significance values.

**Table 5:** Multiple linear regression showing the association between ED types and CMRFs.

Risk factors	AN			BN			BED		
	B	95% CI	*P* value	B	95% CI	*P* value	B	95% CI	*P* value
FBG	**−2.5**	**0.962–4.104**	**0.01** ^*∗*^	**−2.9**	**0.992–4.136**	**0.01** ^*∗*^	0.136	0.914–1.288	>0.05
Ferritin	0.377	1.016–1.298	>0.05	0.555	0.433–0.969	>0.05	0.276	0.4154–0.948	>0.05
Haemoglobin	**−3.46**	**0.943–5.241**	**0.03** ^*∗*^	**−3.77**	**0.983–5.326**	**0.02** ^*∗*^	**−4.199**	**0.954–5.748**	**0.01** ^*∗*^
Albumin	**−3.89**	**0.998–5.321**	**0.02** ^*∗*^	**−3.56**	**0.988–5.443**	**0.04** ^*∗*^	**−5.382**	**0.858–5.558**	**0.05** ^*∗*^
HDL-C	0.146	1.122–1.451	>0.05	**−3.245**	**1.213–3.909**	**0.049** ^*∗*^	**−6.198**	**1.975–7.548**	**0.01** ^*∗*^
TC	1.55	0.873–1.321	>0.05	**7.99**	**2.978–8.414**	**0.02** ^*∗*^	**9.976**	**2.894–11.918**	**0.01** ^*∗*^
LDL-C	1.87	1.063–1.204	>0.05	**5.98**	**1.989–6.314**	**0.01** ^*∗*^	**7.576**	**1.994–8.948**	**0.03** ^*∗*^
TAG	0.327	1.306–1.378	>0.05	**4.678**	**1.421–5.951**	**0.05** ^*∗*^	**8.379**	**1.944–9.351**	**0.02** ^*∗*^
TC: HDL-C	1.36	0.963–1.126	>0.05	**5.78**	**1.954–7.926**	**0.04** ^*∗*^	**9.36**	**2.877–9.431**	**0.01** ^*∗*^
CRP	1.80	0.973–1.92	>0.05	**5.89**	**1.918–6.424**	**0.03** ^*∗*^	**7.973**	**3.890–10.118**	**0.01** ^*∗*^
BMI	**−4.379**	**1.906–4.358**	**0.04** ^*∗*^	**8.955**	**2.533–9.996**	**0.02** ^*∗*^	**10.387**	**3.918–12.948**	**0.01** ^*∗*^
AIP	0.197	1.186–1.978	>0.05	**5.435**	**1.331–6.819**	**0.03** ^*∗*^	**6.992**	**1.987–6.801**	**0.04** ^*∗*^
*R* ^2^	0.621			0.626			0.619		

Multiple linear regression was carried out to analyze the association between ED types, biomarkers, AIP, and BMI. Unstandardized coefficients (B), odds ratios (ORs), and 95% confidence intervals (CIs) were statistically significant at *P* ≤ 0.05^*∗*^ or ≤0.001^*∗∗*^. *R*^2^: coefficient of regression: Unstandardized coefficients; the value of *R*^2^ ranged from 0 to 1. *P* ≤ 0.05^*∗*^, ≤0.001^*∗∗*^. Bold values indicates the significance values.

## Data Availability

The dataset used to support the findings of this study is available on request from the corresponding author.

## Additional Points

*Key Points*. (i) Women with ED had a high risk of cardiovascular diseases. (ii) Women with ED had hypoalbuminaemia, dyslipidemia, anaemia, and high AIP levels. (iii) Blood glucose levels were critically low among women with anorexia nervosa (AN) and bulimia nervosa (BN), but normal among those with binge eating disorders (BEDs). (iv) Saudi women with AN, BN, and BED suffer from multiple cardiometabolic risk factors (CMRFs).
